# Physics-Based Forward Modeling of Ocean Surface Swell Effects on SMAP L1-C NRCS Observations †

**DOI:** 10.3390/s22020699

**Published:** 2022-01-17

**Authors:** Shanka N. Wijesundara, Joel T. Johnson

**Affiliations:** ElectroScience Laboratory, The Ohio State University, 1330 Kinnear Road, Columbus, OH 43212, USA; johnson.1374@osu.edu

**Keywords:** SMAP, backscatter radar cross section, RCS, swell waves, oceans, rough surface scattering, two scale model, electromagnetic modeling

## Abstract

This paper examines the impact of ocean surface swell waves on near-coastal L-band high-resolution synthetic aperture radar (SAR) data collected using the National Aeronautics and Space Administration’s (NASA) Soil Moisture Active/Passive (SMAP) radar at 40° incidence angle. The two-scale model and a more efficient off-nadir approximation of the second-order small-slope-approximation are used for co- and cross-polarized backscatter normalized radar cross-section (NRCS) predictions of the ocean surface, respectively. Backscatter NRCS predictions are modeled using a combined wind and swell model where wind-driven surface roughness is characterized using the Durden–Vesecky directional spectrum, while swell effects are represented through their contribution to the long wave slope variance (mean-square slopes, or MSS). The swell-only MSS is numerically computed based on a model defined using the JONSWAP spectrum with parameters calculated using the National Data Buoy Center and Wave Watch III data. The backscatter NRCS model is further refined to include fetch-limited and low-wind corrections. The results show an improved agreement between modeled and observed HH-polarized backscatter NRCS when swell effects are included and indicate a relatively larger swell impact on L-band compared to higher radar frequencies. Preliminary investigations into the potential swell retrieval capabilities in the form of excess MSS are encouraging, however further refinements are required to make broadly applicable conclusions.

## 1. Introduction

The generation and dissipation of ocean surface waves at the air–sea boundary layer involves many complex and often non-linear processes at various spatial and temporal timescales [1,2]. These processes induce varying degrees of backscatter normalized radar cross section (NRCS) modulations, which manifest as ocean surface features in scatterometer measurements or synthetic aperture radar (SAR) imagery. Wind-driven waves (“wind-waves”) act as the primary contributor to NRCS modulations, especially at moderate incidence angle geometries ($30{}^{\circ}\leq \theta_{i}\leq 60{}^{\circ}$) where Bragg scattering is dominant [3]. The scattering process is typically assumed to be related to local forcing winds with longer wind waves providing “tilt” effects. Therefore, physics-based backscatter NRCS forward modeling studies typically rely on wind-driven ocean wave spectra to characterize rough surface effects. However, several past studies have shown that the presence of swell waves can impact radar measurements.

Swell effects have long been considered to be important in many ocean models [4]. The distribution of swell waves over global oceans predicted by numerical models such as Wave Watch 3 (WW3; [5]) and European Center for Medium-range Weather Forecasts (ECMWF; [6]) has been investigated in the literature [7,8]. They rely on moment parameters such as significant wave height (SWH) to characterize global swell probabilities, peak swell frequency distributions, and swell indices. Spaceborne observations and in situ measurements have also been used for modeling swell propagation characteristics with an emphasis on determining the locations of swell sources [9,10,11]. The impact of swell waves on ERS-1/2 C-band SAR backscatter NRCS measurements has been investigated through SWH comparisons in [12]. SAR-based SWH retrievals using a neural networks algorithm are compared with National Data Buoy Center (NDBC) buoy measurements to conclude that swell indeed has an impact on C-band scatterometers. Reference [13] further examines the impact of swell on both C- and Ku-band near normal incidence backscatter for multiple polarizations, and finds swell contributions to be relatively more important at low wind speeds. Although these studies provide insight into the impact of swell waves, continued analyses into the effects of swell on radar oceanography are well motivated to characterize its effects.

High-resolution spaceborne SAR imagery, enabled by significant advancements in ocean scatterometry, has emerged as an invaluable tool in ocean surface observations [3,14,15,16,17,18]. Systems operating at L-, C-, or X-band frequencies with swath widths ranging from 100–500 km and spatial resolutions of 10–100 m (for example, the ERS-1/2, Envisat, Radarsat 1/2, and PALSAR missions) have shown the capabilities of SAR systems in sensing oceanic phenomena [19]. While SAR imagery is largely unaffected by rain and cloud conditions, more so at L-band frequencies, the long repeat periods, often ranging from weeks to months, have challenged the use of SAR measurements in many practical applications.

**Figure 1:**
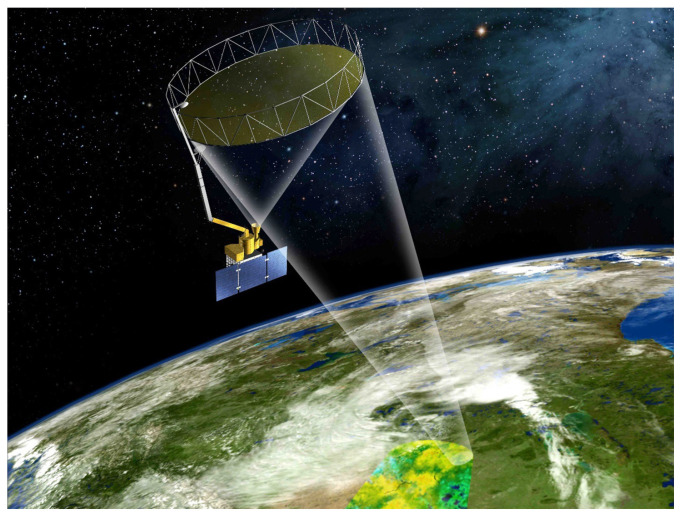
An artist’s rendering of SMAP observations from orbit. Image credit: NASA Jet Propulsion Laboratory (JPL) https://smap.jpl.nasa.gov/resources/59/smap-taking-data-from-orbit, Date Accessed: 15 May 2021.

On 31 January 2015, the National Aeronautics and Space Administration (NASA) launched the Soil Moisture Active/Passive (SMAP) L-band mission to make significantly improved soil moisture and freeze/thaw observations of global land surfaces (see Figure 1) [20]. In addition to soil moisture products, the SMAP radar also provided 1 km high resolution SAR radar data (“L1-C” data) over near-coastal regions at a global revisit rate of 2–3 days during its operational period. This paper investigates the impact of swell waves on SMAP L1-C backscatter radar data through physics-based forward modeling of SMAP radar observations. We use a combined wind and swell ocean model (instead of a wind-only model) and represent the impact of swell as an excess contribution to the surface slope variance that increases “tilting” effects in the two-scale theory of sea surface backscattering. Characterizing the impact of swell waves on SMAP radar data can be directly applied to introduce potential corrections to SMAP-based model functions, which may lead to refined wind retrievals. Furthermore, insights gained from such a study further the understanding of the impact of swell waves on L-band backscatter NRCS, and can be leveraged to observe/model other ocean features that are visible in high-resolution L-band SAR imagery. A limited-scope preliminary version of the work presented in this paper can be found in [21].

The remainder of this paper is organized as follows: Section 2 reviews models for sea surface backscattering, including the two-scale theory and the second-order small slope approximation. The key components of each model are discussed with an emphasis on distinguishing between wind and swell effects. A combined wind and swell ocean model is then presented with the impact of swell represented as an additional long wave tilt effect. Section 3 outlines various data sources used within the modeling study, their utility, and associated challenges and limitations. It also provides a broader overview of the SMAP instrument, mission, capabilities, and available data. Additional details on SMAP data processing and model predictions are then discussed in Section 4. Section 5 discusses the swell-only 2D directional ocean spectra that are used for calculating swell-only slope contributions. Results and additional model refinements are discussed in Section 6, while Section 7 provides concluding remarks.

## 2. Backscatter NRCS Model

### 2.1. Two-Scale (Composite) Model (TSM)

**Figure 2:**
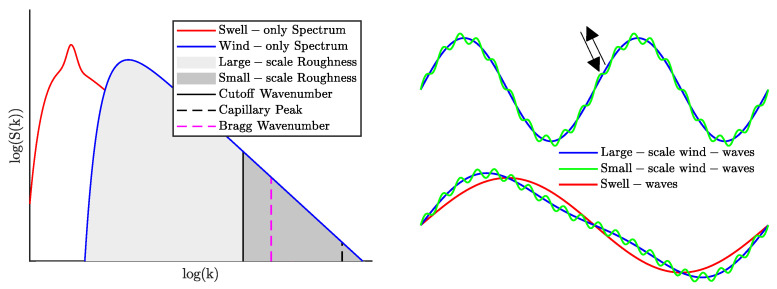
Notional ocean spectrum and resulting ocean waves. The (**left**) panel compares wind-only (blue) and swell-only (red) wave spectra. The wind-only spectrum is divided along the cutoff wavenumber $k_{c}$ to distinguish large- and small-scale roughness contributions. The Bragg and capillary wavenumbers are also shown for reference. The (**right**) panel shows notional wind waves (**top**) and wind and swell waves (**bottom**), while arrows indicate incident and backscattered EM waves.

The two-scale model, also known as the composite surface theory, is a well-studied technique used for modeling microwave backscatter from multi-scale rough surfaces [14,22,23,24]. According to the TSM, radar backscatter NRCS at moderate incidence angles such as SMAP’s $\theta_{i}=40{}^{\circ}$ is dominated by Bragg waves. Bragg scattering, which is mathematically described by the small-perturbation method (SPM), is a resonant reflection of sea surface waves with wavenumber $K_{b}=2k_{0}\sin\theta_{i}$, where $k_{0}=2\pi /\lambda$ in rad/m and λ in meters are electromagnetic wavenumber and wavelength, respectively [3,25]. For SMAP, the Bragg wavelength is approximately $\Lambda_{b}=18.5$ cm (i.e., $K_{b}=33.93$ rad/m) which remains firmly within the “gravity” wave portion of the sea spectrum as opposed to shorter capillary waves. The presence of ocean waves with much longer wavelengths within the SMAP 1 km resolution cell causes “tilting” of Bragg waves as shown in Figure 2. The TSM accommodates both small and large waves (i.e., roughness scales) by defining a cutoff wavenumber $k_{c}$ such that the overall predicted backscatter due to cumulative scattering from tilted Bragg waves is [14]:

(1) $$\sigma_{sea}^{0}{\left(\theta_{i}\right)}_{\hat{i}\hat{j}}=\int_{-\infty }^{\infty }d\left(\tan\psi \right)\int_{-\infty }^{\infty }d\left(\tan\delta \right)\sigma_{\hat{i}\hat{j}}^{0^{\prime }}\left(\theta_{i}^{\prime }\right)F\left(\tan\psi ,\tan\delta \right)W\left(2k_{0}\alpha ,2k_{0}\gamma \sin\delta \right),$$

where ψ and δ are in-plane and out-of-plane tilt angles in radians, respectively, $\gamma =\cos(\theta_{i}+\psi )$, $\alpha =\sin(\theta_{i}+\psi )$, and $\hat{i}$ and $\hat{j}$ represent receive and transmit polarizations, respectively. The backscattered NRCS from a tilted surface is computed using NRCS coefficients $\sigma^{0^{\prime }}\left(\cdots \right)$ evaluated at the local incidence angle of the tilted surface $\theta_{i}^{\prime }=\cos^{-1}\left(\gamma \cos\delta \right)$ and projected onto the “global” radar polarization basis from the “local” basis of the tilted surface. The 2D sea surface spectrum W(⋯) within the integral is evaluated at the Bragg wavenumber that corresponds to the local incidence angle. The cumulative sea surface backscatter NRCS $\sigma_{sea}^{0}$ is then computed by integrating over the long wave slope probability density function (PDF) F(⋯). It is modeled as a zero-mean Gaussian PDF (i.e., Cox and Munk correction term in [26] set to 1):

(2) $$F\left(s_{u}^{\prime },s_{c}^{\prime }\right)=\frac{1}{2\pi s_{u}s_{c}}e^{-0.5\left[{\left(s_{u}^{\prime }/s_{u}\right)}^{2}+{\left(s_{c}^{\prime }/s_{c}\right)}^{2}\right]},$$

where $s_{u}^{\prime }=\tan\psi$ and $s_{c}^{\prime }=\tan\delta$. The up- and cross-wind mean-square slopes (MSS) $s_{u}^{2}$ and $s_{c}^{2}$ are determined from:

(3) $$\left\{s_{u}^{2},s_{c}^{2}\right\}=\int_{0}^{2\pi }\int_{0}^{k_{c}}k^{2}\left\{\cos^{2}\phi ,\sin^{2}\phi \right\}S\left(k,\phi \right)kdkd\phi .$$

Note that the PDF is evaluated at up- and cross-wind slope values that correspond to the in- and out-of-plane tilts following a coordinate rotation. Additionally, S(k,ϕ) is the polar coordinate representation of $W(k_{x},k_{y})$ in Equation (1) that may be augmented by the presence of swell. Cutoff wavenumber values in the range $k_{c}=\left[k_{0}/3,k_{0}/2\right]$ have been recommended in the literature—we use $k_{c}=k_{0}/2$ [24,27,28] in what follows. Note that TSM model predictions weakly depend on this parameter.

Although the preceding discussion only considers Bragg scattering, non-Bragg scattering associated with wave breaking effects also contribute to the ocean surface roughness. The VV polarization is typically dominated by Bragg scattering while HH and HV/VH may contain relatively greater non-Bragg contributions. These effects have been observed and modeled under various frequencies, wind speeds, and incidence angles in the literature [29,30,31]. While the impact of breaking waves has been shown to be important in C-band and higher frequencies, the magnitude of L-band breaking wave contribution has been found to be minimal [31]. Therefore, the model considered in this work does not include any impact due to breaking waves.

### 2.2. Small Slope Approximation for Cross-Polarization

TSM predictions have been shown to be reliable in most situations for co-polarized backscatter at moderate incidence angles. However, TSM predictions of cross-polarized backscatter are challenged by the model’s use of the first order SPM (SPM1) for the local surface backscatter in Equation (1) as they are only predicted to be a result of out-of-plane tilt effects (i.e., δ≠0) [14,25]. In general, cross-polarized scattering from rough surfaces can involve higher-order scattering effects. Although the second-order small slope approximation (SSA2) model captures higher-order scattering and surface tilt effects, its utility for large-scale simulation studies is constrained by the computational complexity [32]. Therefore, a more efficient off-nadir SSA2 cross-polarized high frequency approximation proposed in [33] is examined here:

(4) $$\sigma_{hv}^{0}\approx 4\pi {\left|G\right|}^{2}\cot^{2}\theta_{i}Q_{H}^{4}W\left(Q_{H}\right)s_{x}^{2},$$

where *G* and $Q_{H}$ are described in [33]. The cross-plane MSS is

(5) $$s_{x}^{2}=\int_{0}^{2\pi }\int_{0}^{k_{0}}k^{2}\sin^{2}\phi S\left(k,\phi \right)kdkd\phi ,$$

in which the cutoff wavenumber is defined as the electromagnetic wavenumber, and the coordinates in the integration are aligned with the radar look direction. More generally, Equation (4) approximates cross-polarized backscattering as an easily evaluated product of the surface curvature spectrum evaluated at the Bragg wavenumber ($Q_{H}^{4}W\left(Q_{H}\right)$), the cross-plane slope variance, and functions of permittivity (*G*) and incidence angle.

### 2.3. 2D Wind-Driven Sea Surface Spectrum

Wind-driven sea surface spectra $S_{w}\left(k,\phi \right)$ describe the wind-generated wave energy distribution over two-dimensional frequency and angular space. Many theoretical, empirical, and semi-empirical spectrum models have been proposed over the years to parameterize this wave energy distribution to varying degrees of accuracy [34,35,36,37,38]. A general comparison of such models is beyond the scope of this study (the reader is referred to [39] and sources therein for additional information), however, it is noted that predicted NRCS dependence on wind speed is sensitive to the spectrum choice [40].

**Figure 3:**
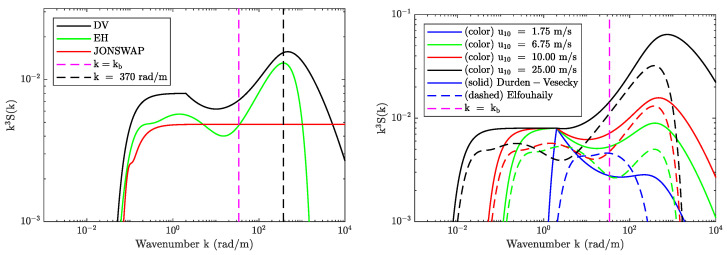
Comparison of wind-driven curvature spectra. (**Left panel**) compares Durden–Vesecky (DV), Elfouhaily (EH), and JONSWAP spectra at $u_{10}=10\;m/s$. (**Right panel**) compares Durden–Vesecky and Elfouhaily curvature spectra over different wind speeds. SMAP Bragg wavenumber $k_{b}=33.93\;\mathrm{rad}/m$ is also shown using a magenta dashed line in both figures.

The left panel in Figure 3 compares the fully-developed Durden–Vesecky (DV-[36]), Elfouhaily (EH-[37]), and JONSWAP ([35]) curvature spectra at the SMAP Bragg wavelength (indicated using the vertical dashed magenta line) for $u_{10}=10\;m/s$. While the JONSWAP and EH spectra show similar magnitudes, they differ considerably from the DV spectrum. Furthermore, the spectra in the vicinity of the Bragg wavenumber differ based on their description of the capillary peak and other factors.

The right panel in Figure 3 compares the DV (solid curves) and EH (dashed curves) spectra as a function of wind speed (blue, green, red, and black colors). A key observation made here is that while the DV spectrum evaluated at the Bragg wavenumber monotonically increases with wind speed, the same does not hold for the EH spectrum. These differences are related to how the spectrum is defined in [37], where the separation of two wave spectra regimes in the short wave domain corresponds approximately to a wind speed of $u_{10}=6.75$ m/s. This effect is also present in other spectrum models (such as the revised Kudryavstev spectrum in [38]) and leads to NRCS predictions with a local minima at the long/short wave partition wind speed similar to the L-band predictions shown in Figure 15a of [39]. In contrast, geophysical model functions (GMF) based on L-band observations at moderate incidence angles support a monotonic increase in NRCS versus wind speed (see SMAP L1B at $\theta_{i}=40{}^{\circ}$ in [41], Aquarius beam-2 at $\theta_{i}=38{}^{\circ}$ in [42], PALS at $\theta_{i}=45{}^{\circ}$ in [43], and PALSAR at $\theta_{i}=40{}^{\circ}$ in [44]).

Due to this behavior, the Durden–Vesecky sea surface spectrum model is applied in this work. The spectrum has the form:

(6) $$\begin{array}{ccc} S_{w}\left(k,\phi \right) & = & \frac{1}{k}S_{w}\left(k\right)\Psi_{w}\left(k,\phi \right) \end{array}$$

(7) $$\;\begin{array}{ccc} & = & k^{-4}\Psi_{w}\left(k,\phi \right)\left\{\begin{array}{cc} b_{0}e^{-\beta {\left(k_{m}/k\right)}^{2}} & \;k<2\\ a_{0}{\left(\frac{bku_{*}^{2}}{g_{*}}\right)}^{a\log_{10}\left(k/2\right)} & \;k\geq 2, \end{array}\right. \end{array}$$

where $S_{w}\left(k\right)$ and $\Psi_{w}\left(k,\phi \right)$ are the 1D spectrum in $m^{3}$ and 2D spreading function, respectively. The wind speed in m/s at 19.5 m above mean sea level (MSL) is defined as $u_{19.5}$, while the friction velocity in m/s is denoted using $u_{*}$. Variables a=0.225, b=1.25, and β=0.74 are empirically derived parameters; $k_{m}=g/u_{19.5}^{2}\;\mathrm{rad}/m$ , $g_{*}=g+\gamma k^{2}\;m/s^{2}$, $\gamma =7.25\times 10^{-5}\;m^{3}/s^{2}$, and $g=9.81\;m/s^{2}$. Constant $a_{0}$ is set to 0.008 as proposed in [45], which is double that of the original reference [46]. Similarly, $b_{0}=a_{0}$ is also amended with

(8) $$b_{0}=a_{0}e^{\beta {\left(k_{m}/2\right)}^{2}}$$

to ensure continuity at the k=2 rad/m boundary [45]. The spreading function $\Psi_{w}\left(k,\phi \right)$ is modeled as unity plus a wavenumber and wind speed dependent amplitude of cos2ϕ. Note that $S_{w}\left(k\right)$ diminishes rapidly for $k<k_{p}$ where $k_{p}$ is the wavenumber of the 1D spectrum peak. As a result, lower wavenumber bounds in Equations (3) and (5) are typically changed from 0 to $g/3u_{19.5}^{2}\approx 0.5k_{p}$. Components of Elfouhaily and JONSWAP spectra will also be used in subsequent sections for model refinements and swell spectrum modeling.

### 2.4. Combined Wind and Swell Model

Unlike wind-driven seas where local wave conditions are determined by local forcing winds, local swell conditions are influenced by propagation of many remote wind-generated waves into the observational area. The propagation effects are akin to a low-pass filtering process where damping of higher frequencies occurs as the wave propagates further away from its origin retaining waves with smaller temporal frequencies (larger periods) [5]. Therefore, wind and swell waves within SMAP 1 km resolution cells are assumed to be independent, uncorrelated Gaussian processes such that

(9) $$S\left(k,\phi \right)=S_{w}\left(k,\phi \right)+S_{s}\left(k,\phi \right)$$

with second-order moments (MSS)

(10) $$\begin{array}{ccc} s_{u}^{2} & = & s_{u,w}^{2}+s_{u,s}^{2} \end{array}$$

(11) $$\begin{array}{ccc} s_{c}^{2} & = & s_{c,w}^{2}+s_{c,s}^{2} \end{array}$$

(12) $$\;\begin{array}{ccc} s^{2} & = & s_{u}^{2}+s_{c}^{2}=s_{w}^{2}+s_{s}^{2}. \end{array}$$

where the subscripts *w* and *s* represent wind and swell spectra/MSS, respectively, and the swell spectrum is written in the up-cross-wind coordinate system. In Equations (9)–(12), the impact of swell on overall backscatter NRCS is modeled as an excess slope contribution that leads to additional tilting in the TSM as shown in Figure 2. Note that the MSS definitions in Equations (3) and (5) remain applicable to swell spectra. In general, multiple swell systems with different peak wave periods and mean wave directions could be present within a 1 km resolution cell such that

(13) $$S_{s}\left(k,\phi \right)=\sum_{n}S_{s,n}\left(k,\phi \right)$$

where each unique swell system identified using index *n* can, as in Equation (6), be represented as a product of a spreading function and a 1D wavenumber spectrum. The proposed representation of swell effects as an excess slope assumes the availability of swell-only or total (including wind and swell) MSS information which, for example, could be made available from numerical wave models such as WaveWatch III or the ECMWF. As the MSS is not typically reported in wave model outputs, an alternate method could compute the MSS using Equations (3) and (5) if a complete 2D swell spectrum (or information that can be used to approximate the 2D spectrum) is available.

## 3. Data Sources

### 3.1. SMAP Mission and L1-C SAR High-Resolution Radar Data

The SMAP satellite hosts both a radar that operates at a tunable frequency near 1.26 GHz and a microwave radiometer that operates at 1.41 GHz. Both instruments share a 6 m mesh reflector antenna that rotates at 14.6 revolutions per minute (RPM) about its nadir axis (see Figure 1) to produce a conically-scanned beam with a surface incidence angle of $\theta_{i}=40{}^{\circ}$ and a swath width of 1000 km, which enables global coverage in 2–3 days. The radar is capable of making both co- and cross-polarized measurements (HH, VV, and HV), which are further discriminated based on the instrument look direction (forward, fore; backward, aft) and orbit direction (ascending, A; descending, D). Detailed descriptions of the SMAP mission, radar data processing, and data formats can be found in [20,47,48].

**Figure 4:**
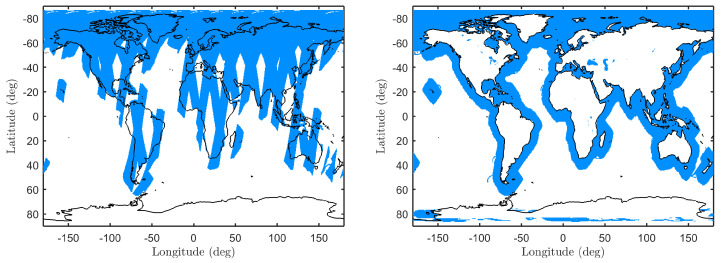
SMAP L1-C global coverage (HH-polarization at fore look direction) highlighted in blue. (**Left**): Daily global coverage on 30 May 2015 (emphasizes individual SMAP passes). (**Right**): Composite near-coastal coverage mask for all available data.

The SMAP radar produces two backscatter NRCS data products: (1) a continuous “low-resolution” (approximately 30 km) L1-B product, and (2) a “high-resolution” (approximately 1 km) L1-C product over land surfaces and near-coastal regions. Figure 4 shows example HH fore L1-C coverage highlighting individual passes over one day, and a composite near-coastal coverage mask for all available data. Unfortunately, the SMAP radar experienced a failure and ceased operation on 7 July 2015. Nevertheless, the data gathered prior to this event provides a unique opportunity to investigate the impact of swell waves on L-band high-resolution ocean backscatter NRCS measurements.

This study utilizes all available SMAP L1-C radar data as summarized in Table 1. The dataset spans from 13 April to 7 July 2015 and consists of 2326 data files. The analysis examines SMAP forward look direction backscatter data in all polarizations, as the forward look case has greater global near-coastal coverage compared to the aft look case. In addition to near-coastal data, L1-C collections over an extended portion of the Atlantic ocean were planned, but only two complete passes over this region were possible (orbits 02300 and 02301) prior to the radar failure.

### 3.2. NOAA WW3 Data

Modeling SMAP radar backscatter measurements requires both wind and wave information. A previous analysis considering only wind effects reported a GMF representing an empirical wind speed–NRCS relationship that was used to retrieve ocean surface wind speeds successfully [41]. However, this GMF does not separate wind and wave effects, and is therefore only used as a reference in this paper. SMAP-based wind retrievals are also available through sources such as Remote Sensing Systems (https://www.remss.com/, accessed on 15 November 2021), but the use of such information in this modeling study creates an undesired circular dependency between outcomes and sources. Wind vector information was instead obtained from the National Oceanic and Atmospheric Administration’s (NOAA) operational Global Forecast System (GFS). Wave information was obtained from NOAA’s WW3 operational wave model predictions. It uses a robust spectral partitioning technique referred to as the Wave Spectrum Energy Partitioning (WaveSEP) method [50] to separate wind and individual swell systems and report their contributions using moment parameters as outlined in Table 2 (see [5] for definitions). Moment parameters for the full 2D spectrum are listed under partition 0 (n=0), while contributions are separated into individual wind/swell (n=1) and swell-only (n>1) systems using subsequent partitions. For mixed wind/swell partitions (n=0,1), the wind-fraction $W_{f}$ is used to identify the portion of the partition under the influence of wind; by definition, $W_{f}\equiv 0$ for swell-only partitions. As the combined wind and swell model relies on swell-only MSS values, the information provided in partitions numbered greater than or equal to one are utilized for swell modeling in this paper.

**Figure 5:**
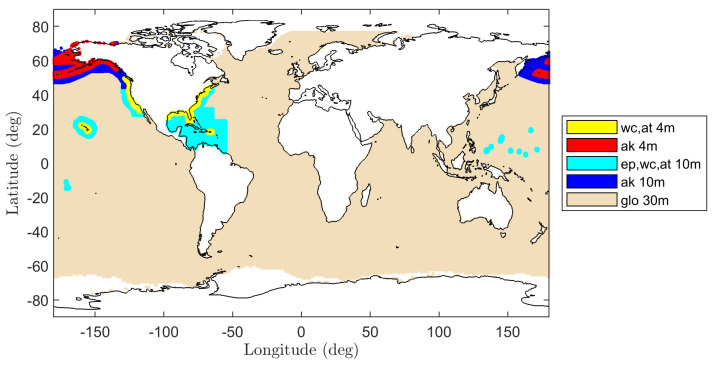
WW3 MultiGrid spatial resolution map; 4, 10, and 30 arc-min regions are shown using yellow/red, light/dark blue, and light-gray colors, respectively. See [51] for additional details.

The combined WW3/GFS wind and wave products are available at 1 hour time increments over three spatial resolutions as shown in Figure 5: (1) 4 arc-min (≈7 km) over coasts of the continental United States, Alaska, Puerto Rico, and Hawaii, labeled wc_4m, at_4m, and ak_4m; (2) 10 arc-min (≈18 km) over 4 arc-min regions and the US territories in the southern pacific labeled wc_10m, at_10m, ak_10m, and ep_10m; and (3) 30 arc-min (≈55 km) globally labeled glo_30m. Although wind and wave predictions at resolutions finer than even 7 km are desirable when predicting SMAP 1 km radar backscatter, the global availability of the WW3/GFS data, especially in the southern hemisphere where prominent swell waves are present, motivates the use of the 30 arc-min wind product, with finer resolution wind products used for additional verification purposes.

### 3.3. NDBC 2D Buoy Spectra

**Figure 6:**
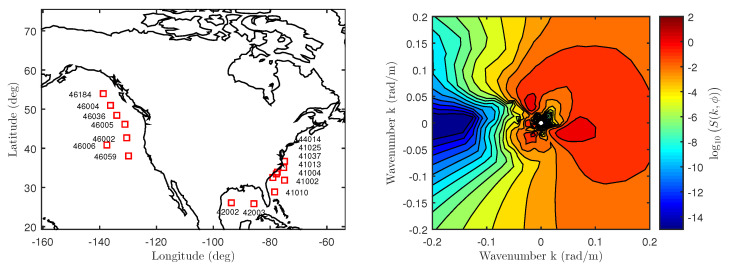
Buoy Data. (**Left**): Locations of the 16 buoys used. The buoy labels in the central Atlantic coast are arranged based on latitude such that top-most buoy corresponds to the top-most buoy label. (**Right**): 2D spectrum of Buoy 46006 on 14 June 2015 at 12:00:00 UTC in the up-cross-wind coordinate system. The associated wind speed is $u_{10}=5.63$ m/s from ϕ=50.3°.

The 1D swell-only spectrum model to be discussed in Section 5.1 utilizes 2D NDBC buoy spectra [52]. They are arranged in a [50 × 36] [frequency × azimuth] grid and are computed every 3 h daily starting at midnight. The 2D directional spectra are estimated using a simpler directional Fourier approach, and do not use advanced methods such as the Maximum Likelihood Method (MEM) or Maximum Entropy Method (MEM) [53]. The azimuth grid is uniformly sampled at every 10° while the frequency grid is non-uniformly sampled between $f_{b,min}=0.0350$ Hz and $f_{b,max}=0.9640$ Hz. This study uses data from 16 buoys over May, June, and July of 2014 and 2015 with locations shown in Figure 6. An example 2D spectrum oriented in the up-cross-wind direction (+x-axis indicates the up-wind direction) is also shown with contributions from wind and multiple swell systems with peak periods between $11\leq T_{p}\leq 20$ s.

## 4. SMAP Data Processing and Wind-Driven Model Predictions

**Figure 7:**
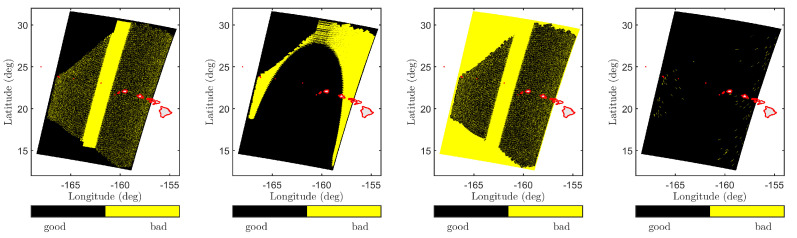
SMAP L1-C quality flags for orbit 01318D on 1 May 2015 over Hawaii. Figures from (**left**) to (**right**) show $K_{p}$ noise, Faraday correction, look-quality, and presence of RFI. In each figure, the black region identifies high-fidelity data, while the yellow region identifies poor quality data. The overall quality mask is a combination of these and other quality flags. See [47] for additional details.

**Figure 8:**
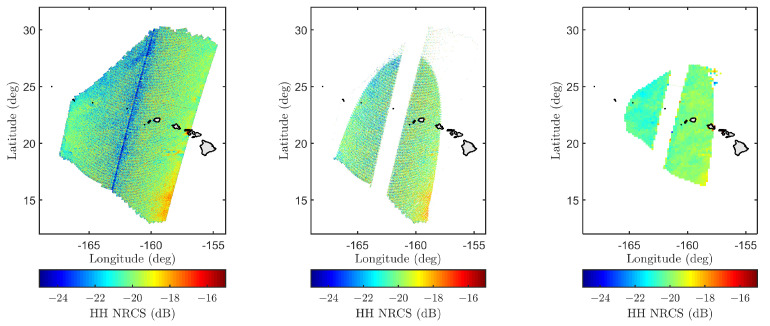
SMAP L1-C HH-polarized backscatter NRCS for orbit 01318D on 1 May 2015 over Hawaii. Figures from (**left**) to (**right**) show original SMAP measurements, high-fidelity measurements retained after applying quality flags, and SMAP NRCS degraded to ep_10m resolution.

SMAP measurements were first screened by applying SMAP and user-defined quality flags. A subset of the SMAP quality flags is outlined in Figure 7 for orbit 1318D on 1 May 2015, over Hawaii. The yellow regions in each figure fail to meet data quality thresholds and the cumulative yellow shaded region is excluded from data. The user-defined quality flags include spatial masks to remove SMAP measurements adjacent to land boundaries and regions containing possible sea ice based on Polar View sea ice data (https://www.polarview.aq/, Date Accessed: 6 November 2018). SMAP L1-C data at 1 km original spatial resolution were then averaged to 4, 10, or 30 arc-min resolutions to match-up with the WW3/GFS dataset as shown in Figure 8. Model comparisons were facilitated by pre-computing a lookup table for wind-only seas over 84 wind speeds and 36 relative azimuth angles:

(14) $$\begin{array}{ccc} u_{19.5,sim} & = & \left\{1,1.25,1.5,\cdots ,21.5,21.75\right\}\;{\mathrm{ms}}^{-1} \end{array}$$

(15) $$\begin{array}{ccc} \phi_{sim} & = & \left\{-170{}^{\circ},-160{}^{\circ},\cdots ,170{}^{\circ},180{}^{\circ}\right\} \end{array}$$

In Equation (14), $\phi_{sim}$ is defined as the relative azimuth between the SMAP mean antenna azimuth projected onto Earth’s surface and the WW3 wind direction in an up-cross-wind coordinate system.

**Figure 9:**
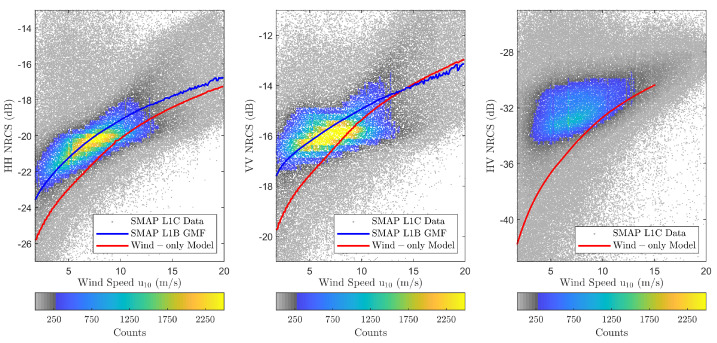
SMAP wind speeds vs. backscatter NRCS scatter density plots. Figures from (**left**) to (**right**) show HH-, VV-, and HV-polarizations. SMAP L1-B GMF (co-pol only) and wind-only model predictions (TSM for co-pol, SSA2 for cross-pol) are also shown.

Figure 9 shows the resulting backscatter NRCS vs. wind speed scatter density plots for each polarization for the entire SMAP dataset. Each plot contains approximately 1.15 million scatter points with the vertical and horizontal axes (NRCS and wind) binned every 0.25 dB and 0.1 ${\mathrm{ms}}^{-1}$, respectively. The empirical GMF from [41] (co-pol only) and the physics-based wind-only backscatter NRCS predictions (TSM for co-pol, SSA2 for cross-pol) are also overlaid to provide direct comparisons. The results show that the co-pol empirical model predictions capture the highest density portions of the scatter plot regions more accurately than the “wind-only” TSM predictions. The under-prediction of the TSM results is greater at lower wind speeds and diminishes with increasing wind speed, and also is greater for the HH polarization. Taken together, these observations suggest the presence of swell that is impacting the backscattered NRCS.

## 5. Calculating Swell-Only Mean-Square Slopes (MSS)

The excess MSS representation presented in Section 2.4 requires swell-only MSS values at all SMAP temporal and spatial locations under consideration. Swell-only MSS is not directly reported by the wave model outputs, but could be computed if a general 2D swell spectrum model is available. The remainder of this section focuses on defining parameters of a general swell spectrum $S_{s}\left(f,\phi \right)$. It is expressed in terms of the temporal wave frequency *f* as

(16) $$S_{s}\left(f,\phi \right)=C_{0}{\bar{S}}_{s}\left(f,\phi \right)=\frac{1}{f}C_{0}{\bar{S}}_{s}\left(f\right)\Psi_{s}\left(f,\phi \right),$$

where ${\bar{S}}_{s}\left(f\right)$ is the 1D swell spectrum with its peak normalized to unity, $\Psi_{s}\left(f,\phi \right)$ is the swell spreading function, and $C_{0}$ is a normalizing constant calculated based on the SWH. Note the frequency-dependent spectrum can be redefined in terms of wavenumber using the deep-water dispersion relationship [35]. Section 5.1 examines the 1D swell spectrum in detail and represents it using the JONSWAP model parameters extended into the swell-dominated regime through a NDBC buoy spectra analysis. Section 5.2 defines the 2D spreading function, while Section 5.3 examines the fidelity of the approximated swell-only 2D spectrum.

### 5.1. 1D Normalized Swell Spectrum

1D swell spectra $S_{s}\left(k\right)$ have been modeled in past studies as a narrow-band Gaussian spectrum, but recent studies have shown a JONSWAP spectrum to be a more accurate substitute [36,54]. In [54], the authors use the mean values for JONSWAP peak shape parameters γ, $\sigma_{a}$, and $\sigma_{b}$ identified for wind-driven spectra in [35] (3.3, 0.07, and 0.09, respectively) to show that the resulting JONSWAP model spectral shape and significant wave height reasonably predicts observed swell spectra with periods $T_{p}\in \left[10,15\right]$ s. Parameter values for γ, $\sigma_{a}$, and $\sigma_{b}$ for other swell period values, however, is not clear in the existing literature. Narrower swell spectra are expected to be associated with larger γ (the “peak enhancement” factor) and smaller $\sigma_{a}$ and $\sigma_{b}$ values (which describe the width of the spectral peak). Although the high-frequency decay for wind-driven spectra is proportional to $f^{-5}$ [35] where *f* is the wave frequency, and [54] uses this rate for swell spectra, the appropriate exponent to use for more general swell spectra is also uncertain.

To obtain swell-only spectra, one has to separate wind-driven spectral components from those driven by swell. Separating swell-seas from wind-seas in the wave spectrum has been thoroughly studied in the literature [55,56,57,58]. A common technique used is to divide the spectrum close to the peak frequency of the wind-driven spectrum $f_{m}$ such that the separation frequency in Hz is

(17) $$f_{s1}=Xf_{m}$$

where *X* is an empirically determined factor identified to be 0.8 and 0.75 in [57,58], respectively, with the latter showing minimum marginal impact if varied in the vicinity of 0.75; this study uses X=0.69.

First, a set of class I, 1D swell-only NDBC buoy spectra with their peaks normalized to unity were compiled (see [54] for class definitions). The resulting normalized swell spectra were then binned based on their peak frequency (50 possible locations based on the NDBC frequency axis), and spectra within each bin were averaged to obtain a set of averaged, normalized swell spectra. The JONSWAP spectrum with its peak normalized to unity was then swept over 48-γ, 30-$\sigma_{a}$, and 30-$\sigma_{b}$ values:

(18) $$\;\begin{array}{ccc} \gamma_{i} & = & \left[1,1.25,1.50,\cdots ,6.75,7.00,8.00,9.00,\cdots ,29.00,30.00\right] \end{array}$$

(19) $$\begin{array}{ccc} \sigma_{a,j} & = & \left[0.01,0.02,\cdots ,0.30\right] \end{array}$$

(20) $$\begin{array}{ccc} \sigma_{b,k} & = & [0.01,0.02,\cdots ,0.30]. \end{array}$$

For each iteration, the root-mean-square error (RMSE) between the JONSWAP and NDBC averaged spectra over a region that immediately surrounds the spectral peak was calculated. Note that confining the RMSE calculation to the spectral peak region deliberately excludes frequencies that define the high-frequency spectrum decay so that the JONSWAP form remains applicable. The RMSE was then minimized to obtain each parameter versus the peak swell frequency.

**Figure 10:**
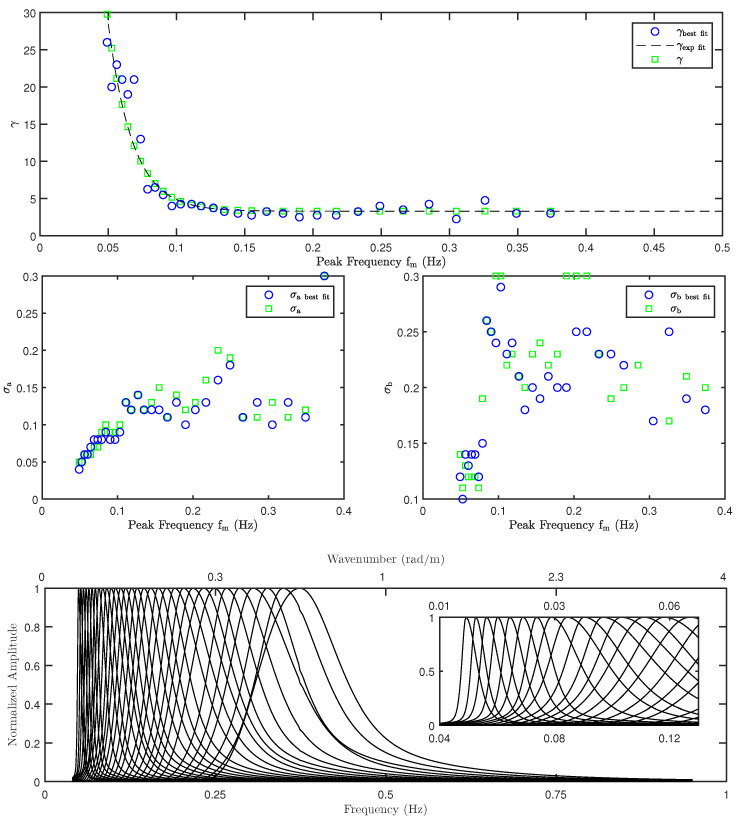
JONSWAP peak shape parameters extended to the swell-dominated region as a function of peak frequency based on NDBC buoy data. Variations in γ, $\sigma_{a}$, and $\sigma_{b}$ are shown in (**top**), (**middle left**), and (**middle right**) figures, respectively. The (**bottom**) figure shows resulting normalized 1D swell spectral shapes ${\bar{S}}_{s}\left(f\right)$. See text for details.

Figure 10 shows the resulting JONSWAP parameters as a function of the peak swell frequency $f_{m}$. In each plot, the optimal parameter values determined through the RMSE minimization described above are shown as blue circles. The minimum peak frequency in the analysis was limited by the minimum NDBC frequency, and no swell-only class I spectra could be identified for $f_{m}>0.375$ Hz using the wind/swell separation technique described earlier. As hypothesized in Section 2, high-frequency damping resulting from swell wave propagation narrows the 1D spectral shape for $f_{m}\leq 0.1$ Hz leading to a sharp increase in the associated γ values. For $f_{m}>0.1$ Hz, γ approaches 3.375, which is in excellent agreement with the mean γ=3.3 reported in [35]. The γ values were then fitted to the dashed curve in the figure as

(21) $$\gamma =a_{0}+a_{1}e^{a_{2}f_{m}}$$

using least-squares minimization that resulted in $a_{0}=3.3$, $a_{1}=408.0$ (both in normalized units), and $a_{2}=-55.7$ s. The green squares in Figure 10 show γ values computed using Equation (21) at the NDBC spectrum frequencies used in the analysis. Similarly, variations in $\sigma_{a}$ and $\sigma_{b}$ are largely in agreement with [35]. The bottom plot finally illustrates the resulting modeled normalized 1D swell-only spectral shapes ${\bar{S}}_{s}\left(f\right)$ at each peak frequency.

### 5.2. Swell Spreading Function

Although the JONSWAP model assumes a $\cos^{2}\phi$ spreading function, we used the $\cos^{2s}$ form suggested in [59]:

(22) $$\Psi_{s}\left(f,\phi \right)=A_{0}\cos^{2s}\left(\left(\phi -\phi_{m}\right)/2\right),$$

due to its consistency with the WW3 model [5]. Here, $\phi_{m}$ is the mean wave direction and

(23) $$s=\frac{2}{\sigma_{\phi }^{2}}-1$$

controls the directional spreading (see (38) in [59]). Note that $\phi_{m}$ and $\sigma_{\phi }$ values are directly available through WW3 partitioned spectra (see Table 2; units converted from degrees to radians). The spreading function is normalized along azimuth such that

(24) $$\int_{0}^{2\pi }\Psi_{s}\left(f,\phi \right)d\phi \equiv 1,$$

which determines the constant $A_{0}$.

### 5.3. 2D Swell Spectrum

The 2D swell-only spectrum can now be modeled using Equation (13) and the spectrum partition information. For each partition, $A_{0}$ in Equation (22) was first identified by enforcing Equation (24) via numerical integration. Similarly, $C_{0}$ was found by enforcing the partition swell-only SWH $H_{s,1/3}$:

(25) $$\;\begin{array}{ccc} H_{s,1/3} & \approx & 4\sqrt{h_{s,rms}^{2}} \end{array}$$

(26) $$\begin{array}{ccc} h_{s,rms}^{2} & = & C_{0}\int_{0}^{2\pi }\int_{f_{sl}}^{f_{su}}{\bar{S}}_{s}\left(f,\phi \right)fdfd\phi , \end{array}$$

where $h_{s,rms}^{2}$ is the swell-only surface height variance in $m^{2}$, and $f_{sl}$ and $f_{su}$ are lower- and upper-cutoff swell frequencies, respectively. It has been experimentally determined that the impact of swell diminishes for wavenumbers that are greater than $k_{su}=2$ rad/m ($f_{su}=0.705$ Hz) [60,61], while the lower-cutoff limit is constrained by the instrument spatial resolution. For SMAP L1-C, it is $f_{sl}=3.95\times 10^{-2}$ Hz, which captures swell wave periods $T_{p}\leq 25.31$ s. $H_{s,1/3}$ is directly available for swell-only partitions (i.e., having wind fraction $W_{f}=0$; see Table 2) and defined for mixed wind/swell partitions as

(27) $$H_{s,1/3}=\sqrt{H_{1/3}^{2}\left(1-W_{f}\right)}.$$

Note that WW3 partitioned peak wave frequencies are not guaranteed to align with NDBC frequencies. This may result in under-sampling of the ${\bar{S}}_{s}\left(f\right)$ spectral peak as it is only defined at NDBC frequencies, leading to incorrect $C_{0}$ values. Therefore, the frequency axis was interpolated to ensure adequate sampling of the spectral peak.

**Figure 11:**
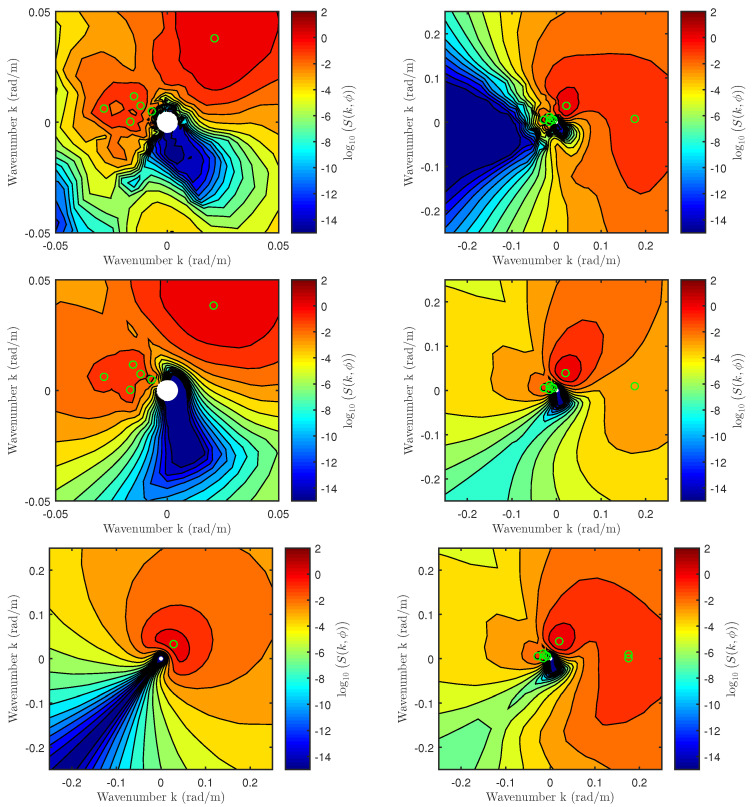
A comparison of NDBC buoy spectra and approximated 2D spectra. Plots shown are as follows: (**top row**): NDBC buoy 46002 spectrum on 2 May 2015 at 00:00:00 UTC ($u_{10}=5.63$ m/s and ϕ=50.3°); (**left**) zoomed-in view; (**right**) zoomed-out view. (**Middle row**): approximated swell-only buoy spectrum using non-zero WW3 partitions; (**left**) zoomed-in view; (**right**) zoomed-out view. (**Bottom row**): (**left**) swell-only spectrum approximated using WW3 0th-partition data; (**right**) middle-right plot with wind contributions added. All plots are in the up-cross-wind coordinate system. See text for more details.

Figure 11 compares measured and approximated 2D spectra for NDBC buoy 46002 on 2 May 2015 at 00:00:00 UTC. The buoy spectrum is shown on the top row where zoomed-in and zoomed-out views are displayed on the left and right plots, respectively. The zoomed-in view highlights swell-only contributions with all six swell-only partition peaks indicated using green circles. The contribution of the wind-dominated partition is highlighted using the zoomed-out view with a green circle in the +x direction (i.e., all spectra shown are in the up-cross-wind coordinate system). The *swell-only* 2D spectrum approximated using non-zero WW3 partitions is shown in the middle row with zoomed-in and zoomed out views on the left and right, respectively. The resulting spectrum appears to capture the buoy swell peaks reasonably both in magnitude and direction. The zoomed out view only shows the fractional swell contribution captured by the wind partition (no wind contributions are shown). For comparison purposes, the bottom right plot shows the approximated *wind and swell* spectrum, which compares favorably with the buoy spectrum shown in the top-right plot. In contrast, the *swell-only* spectrum approximated using the zeroth-partition data (not used in this study) is only able to capture the primary swell direction and magnitude.

Although the approximated spectrum captures buoy swell magnitudes and directions favorably, a clear difference exists in the down-wind direction where the approximated spectrum predicts magnitudes that are much larger than those measured by the buoy. This discrepancy is primarily evident in the swell-only 2D spectrum (middle row), while the inclusion of wind partition information (bottom right) only has a minimal impact in the down-wind direction. Two sources for these differences are possible: (1) the high-frequency decay rate of ${\bar{S}}_{s}\left(f\right)$, and (2) the $\cos^{2s}$-form of $\Psi_{s}\left(f,\phi \right)$. In both cases, the swell-only model assumes parameter values derived based on wind-driven spectra, but a more rapid high-frequency decay rate than $f^{-5}$ and/or a narrower spreading function may be warranted. These investigations are beyond the scope of the current study; note that the differences obtained are at a level exponentially smaller than the peak swell contributions, which are expected to dominate the MSS values of interest.

To account for swell impact, the forward model lookup tables were extended using 31 additional excess slopes in the up-cross-wind directions

(28) $$\begin{array}{ccc} s_{u} & = & s_{u,w}+m\Delta s_{u} \end{array}$$

(29) $$\begin{array}{ccc} s_{c} & = & s_{c,w}+n\Delta s_{c} \end{array}$$

(30) $$\;\begin{array}{ccc} \left\{m,n\right\} & \in & \left\{0,1,2,\cdots ,30,31\right\} \end{array}$$

(31) $$\;\begin{array}{ccc} \left\{\Delta s_{u},\Delta s_{c}\right\} & = & 0.005 \end{array}$$

for a total of 1024 swell conditions (including no swell for m=n=0). The resulting wind speed, relative azimuth, and up-cross-wind swell-only excess slopes lookup table was then used to produce forward model backscatter NRCS predictions under various conditions.

## 6. Results, Additional Refinements, and Discussion

**Figure 12:**
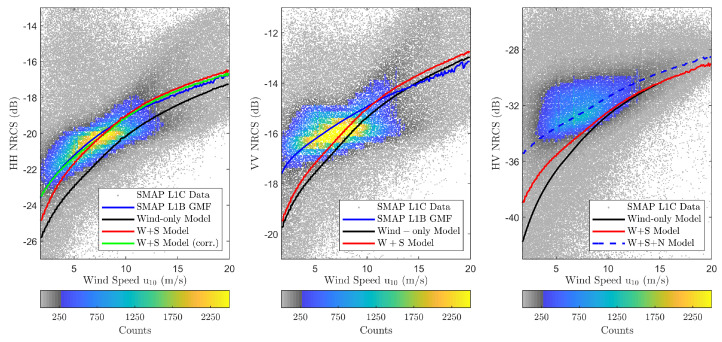
Combined wind and swell model (W+S) backscatter NRCS predictions vs. wind speed shown using red curves. Figures from (**left**) to (**right**) show HH- and VV-polarizations. Corrected W+S model predictions are shown using green curves. Uses the same plot configuration used in Figure 9.

Figure 12 repeats the results of Figure 9 including the combined wind and swell (W+S) model predictions. The impact of swell is clearly visible in HH-polarization where a larger increase in NRCS over wind-only predictions can be observed. VV-polarization shows a reduced impact due to its lack of sensitivity to tilt effects. The cross-polarized results indicate some responsiveness to swell under low wind speeds, but the −38 dB measurement noise floor (see [47]) makes detailed interpretation difficult. Generally, the model predictions are consistent with the discussion in Section 1, increasing confidence in the backscatter model described in Section 2. Based on these observations, the remainder of this paper focuses on characterizing swell effects using the HH-polarization.

Although HH-polarized results compare favorably with the GMF, the combined model shows a larger difference from the GMF predictions at low wind speeds. As discussed in Section 2, the surface roughness due to local winds is captured by the wind model, while swell provides an additional source of Bragg wave tilting. We apply the low-wind correction described below to the modified DV spectrum to achieve improved NRCS predictions under low wind speeds. The combined model also slightly over-predicts the GMF at high wind speeds, potentially due to fetch-limitations in the near-coastal regions considered. Therefore, the fully-developed DV spectrum is further modified to accommodate fetch limited seas using [37].

### 6.1. Fetch-Limited Seas

**Figure 13:**
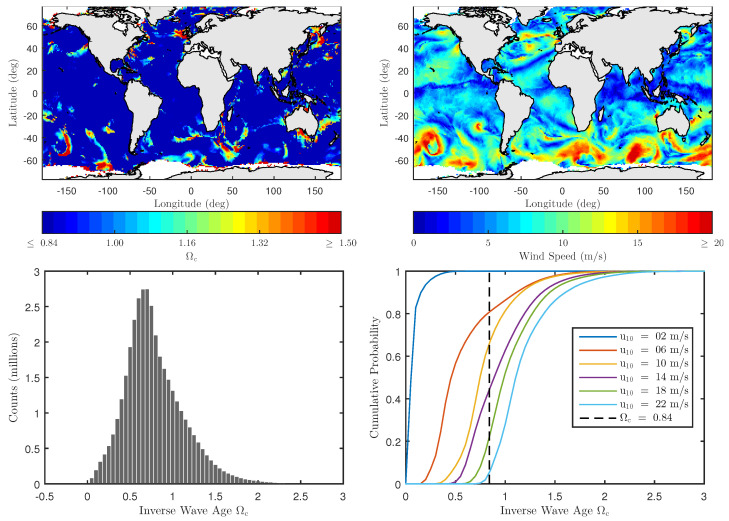
The WW3 inverse wave age $\Omega_{c}$ and wind speed on 1 May 2015, at 00:00:00 UTC at glo_30m spatial resolution are shown on (**left**) and (**right**) plots of the (**top row**), respectively. The distribution of $\Omega_{c}$ computed using Equation (32) for $|\phi_{m}-\phi_{w}|\leq 20{}^{\circ}$ for all L1_C data files in Table 1 is shown in the (**bottom left**) plot. The $\Omega_{c}$ dependence on wind speed is shown in the (**bottom right**) plot using cumulative distribution functions for multiple wind speeds.

Fully-developed wind-driven spectra assume adequate fetch and wind durations that achieve an equilibrium in wave-inducing and restoring forces: this is seldom achieved in reality [1,35]. The fetch requirements defined in [35] suggest that achieving such conditions becomes increasingly challenging for higher wind speeds. Near-coastal regions are further challenged due to geographical constraints and may result in seas that are not fully-developed even at low wind speeds. The Elfouhaily spectrum in [37] models this behavior using the inverse wave-age parameter $\Omega_{c}$

(32) $$\Omega_{c}=\frac{u_{10}}{c_{p}}\cos\bar{\phi },$$

where $c_{p}$ is the wave phase-speed and $\bar{\phi }$ is the relative angle between the wind and dominant wave directions at the spectral peak. Figure 13 illustrates an inverse wave-age map computed using WW3 partitioned spectrum data for1 May 2015, at 00:00:00 UTC at glo_30m spatial resolution; the corresponding wind speed map is also shown for reference. The results show the existence of fetch-limited ocean conditions primarily over high wind and/or near-coastal regions. The histogram on the bottom-left plot further examines the $\Omega_{c}$ distribution for $|\bar{\phi }|<20{}^{\circ}$ over the entire L1-C dataset. It peaks in the vicinity of $\Omega_{c}=\left[0.7,0.8\right]$ and is consistent with the $\Omega_{c}=0.84$ fully-developed sea definition in [37]. It also shows that $\Omega_{c}$ values in the [0.84,2] range are also common. The dependence of $\Omega_{c}$ on wind speed is further explored in the bottom-right plot where $\Omega_{c}$ cumulative distribution functions (CDF) for multiple wind speeds are shown. Under very low wind speeds, for example $u_{10}=2\;{\mathrm{ms}}^{-1}$, nearly all the data can be well-approximated using a fully-developed model, while only 6% appears fully developed for $u_{10}=22\;{\mathrm{ms}}^{-1}$.

The effects of fetch-limited seas on the forward model were predicted using seven additional $\Omega_{c}$ values beyond the 0.84 value originally used:

(33) $$\Omega_{c}=\left\{1.00,1.16,1.48,1.96,2.92,4.04,5.00\right\}.$$

These values span the $\Omega_{c}$ domain in [37] with more frequent sampling for $\Omega_{c}<2$. The expanded look-up table is then used in terms of wind speed, relative azimuth, up-cross-wind swell-only excess slopes, and wave-age to make backscattered NRCS predictions.

### 6.2. Durden–Vesecky Spectrum Modifications

To incorporate model refinements described above, the fully developed Durden–Vesecky spectrum in Equation (7) was modified to include fetch-limited and low-wind correction terms. As fetch-limitations primarily affect the peak of the spectrum, the $k_{m}$ parameter (see Section 2.3) is redefined as

(34) $${\tilde{k}}_{m}=\frac{g}{u_{19.5}^{2}}0.84^{-2}\Omega_{c}^{2}$$

such that Equation (7) is recovered under fully-developed seas. The piece-wise form of Equation (7) models the wind-driven peak using an exponential term for k<2 rad/m, while the function defined over k>2 rad/m captures the capillary peak. The underlying assumption that the wind-driven peak does not cross k=2 rad/m is now rendered false by Equation (34), which necessitates the redefinition of the boundary as

(35) $${\tilde{k}}_{n}=\left\{\begin{array}{cc} {\tilde{k}}_{p} & \;{\tilde{k}}_{p}\geq 2\\ 2 & \;{\tilde{k}}_{p}<2, \end{array}\right.$$

where ${\tilde{k}}_{p}$ is the peak of the 1D DV spectrum

(36) $${\tilde{k}}_{p}=\sqrt{\frac{2\beta }{3}}{\tilde{k}}_{m}.$$

To ensure continuity of the piece-wise form across $k={\tilde{k}}_{n}$, $b_{0}$ is redefined such that

(37) $${\tilde{b}}_{0}=a_{0}{\left(\frac{b{\tilde{k}}_{n}u_{*}^{E}}{{\tilde{g}}_{*}}\right)}^{a\log_{10}\left({\tilde{k}}_{n}/2\right)}e^{\beta {\left({\tilde{k}}_{m}/{\tilde{k}}_{n}\right)}^{2}},$$

where ${\tilde{g}}_{*}=g+7.25\times 10^{-5}{\tilde{k}}_{n}^{2}$. The low-wind correction term is introduced by modifying the friction velocity exponent (equal to 2) in Equations (7) and (37) to

(38) $$\tilde{E}=\left\{\begin{array}{cc} 2 & \;u_{*}\geq {\tilde{c}}_{m}\\ \frac{-0.7}{{\tilde{c}}_{m}^{2}}{\left({\tilde{c}}_{m}-u_{*}\right)}^{2}+2 & \;u_{*}<{\tilde{c}}_{m}, \end{array}\right.$$

where ${\tilde{c}}_{m}=0.3616\;{\mathrm{ms}}^{-1}$ corresponds to $u_{19.5}=10\;{\mathrm{ms}}^{-1}$ (or $u_{10}=9.4\;{\mathrm{ms}}^{-1}$) and is motivated by the excellent agreement between combined model predictions and empirical GMF for HH-polarization seen in the vicinity of $u_{19.5}=10\;{\mathrm{ms}}^{-1}$ in Figure 12. Note that this approach is similar to that of [37], with two regimes defined for the friction velocity, either below or above 0.23 m/s ($u_{10}=6.75\;{\mathrm{ms}}^{-1}$). Therefore, the resulting modified DV spectrum is

(39) $$S_{w}\left(k,\phi \right)=k^{-4}\Psi_{w}\left(k,\phi \right)\left\{\begin{array}{cc} {\tilde{b}}_{0}e^{-\beta {\left({\tilde{k}}_{m}/k\right)}^{2}} & \;k<{\tilde{k}}_{n}\\ a_{0}{\left(\frac{bku_{*}^{\tilde{E}}}{{\tilde{g}}_{*}}\right)}^{a\log_{10}\left(k/2\right)} & \;k\geq {\tilde{k}}_{n}. \end{array}\right.$$

**Figure 14:**
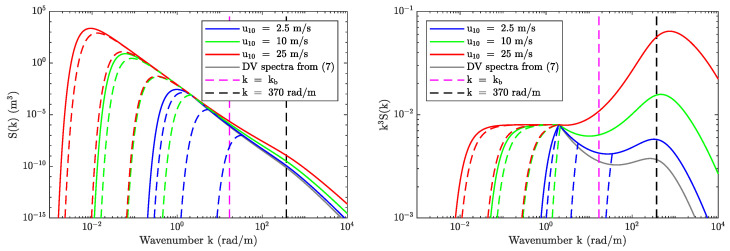
The modified Durden–Vesecky spectra (**left**) and curvature spectra (**right**) for different wind speeds (solid color curves) and $\Omega_{c}$ values (dashed color curves). For each set of three dashed curves, the left-most, middle, and right-most curves represent $\Omega_{c}=1$, $\Omega_{c}=2$, and $\Omega_{c}=5$, respectively. The gray solid curves show corresponding fully-developed DV spectra defined in (7). Note that gray curves for $u_{10}=\left\{10,25\right\}$ m/s are not visible, as (39) fully recovers (7) under those conditions. See text for details.

Figure 14 shows the modified DV spectra and curvature spectra for three different wind speeds and four different $\Omega_{c}$ conditions (fully-developed and three fetch-limited cases). The resulting impact on NRCS can be divided into two categories: (1) those due to changes in the spectral density in the vicinity of the Bragg wavenumber, and (2) those due to a reduction in MSS due to fetch-limited seas. The fully-developed DV spectrum remains unchanged for $u_{19.5}\geq 10\;{\mathrm{ms}}^{-1}$ but results in increased NRCS for $u_{19.5}<10\;{\mathrm{ms}}^{-1}$ due to higher spectral density at the Bragg wavenumber. There is a narrow subset of data where fetch-limited seas at very low wind speeds result in decreased NRCS due to a reduction in spectral density at the Bragg wavenumber. The overall impact of this subset on NRCS predictions is negligible based on the results in Figure 13. In contrast, fetch-limited seas lead to a reduction in MSS over all wind speeds. Based on Figure 13, $\Omega_{c}=2$ can be used as an upper bound to constrain the wave age dependency, however, this still represents a minor perturbation to the spectrum (and to MSS) compared to fully-developed seas. Therefore, a very small reduction in NRCS can be expected at higher wind speeds (where the proportion of fetch-limited seas is greater) while it is negligible at moderate to low wind speeds (where the proportion of fetch-limited seas is smaller). These refinements are expected to increase the combined wind and swell model NRCS predictions over low wind speeds while slightly reducing the NRCS at high wind speeds as hypothesized earlier.

### 6.3. Results and Discussion

The green curve in Figure 12 shows the combined wind and swell model predictions with additional model refinements at glo_30m resolution as a function of wind speed. The results show a significant HH backscatter NRCS model prediction improvement over low wind speeds, while the inclusion of fetch-limited seas decreases the overall NRCS overestimation over high wind speeds. Although the low-wind correction term also resulted in improved VV-backscatter NRCS model predictions (not shown), the VV model predictions still have larger differences with GMF predictions due to the lack of sensitivity to tilt effects.

**Figure 15:**
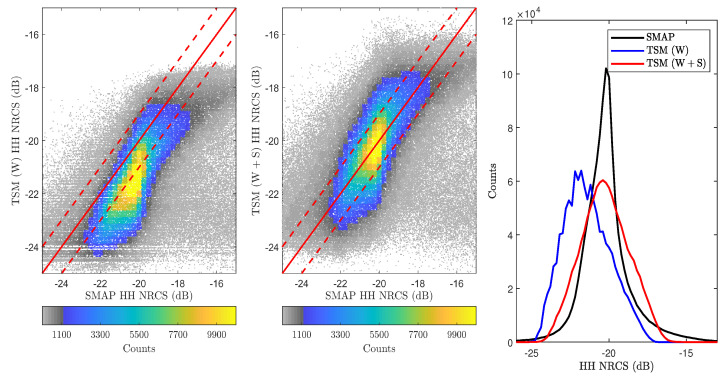
HH-polarized backscatter NRCS model predictions for all L1-C data at glo_30m resolution. Plots from (**left**) to (**right**): SMAP vs. wind-only, SMAP vs. wind and swell, and distributions of NRCS data.

Figure 15 provides scatter density plots that compare SMAP vs. model predictions for the entire L1-C dataset. The percentage of predictions within ±1 dB of SMAP measurements increases from 39% for the wind-only model to 65% for the combined wind and swell model with the largest gains observed at low wind speeds. The combined model increases the average HH-polarized NRCS by approximately 2–2.5 dB to closely match the mean of the SMAP distribution. The wider distribution of the model predictions as compared to measurements is noted as an item warranting further study.

**Figure 16:**
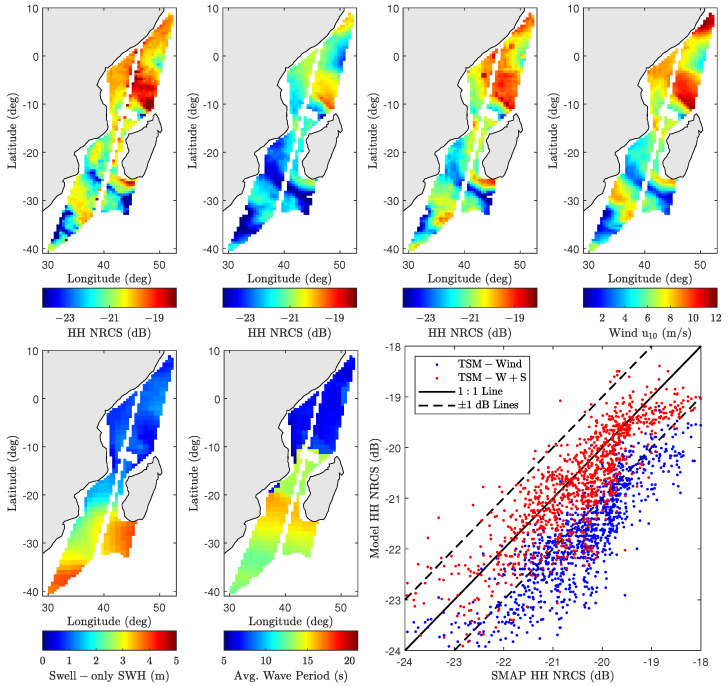
SMAP vs. model backscatter NRCS comparison for pass 02260_D on 5 July 2015, at 02:32:47 UTC near Madagascar at glo_30m resolution. (**Top row**) from (**left**) to (**right**): backscatter NRCS for SMAP, wind-only model, wind and swell model, and wind speed. (**Bottom row**) from (**left**) to (**right**): swell-only SWH, average wave period, measured vs. modeled scatter data.

**Figure 17:**
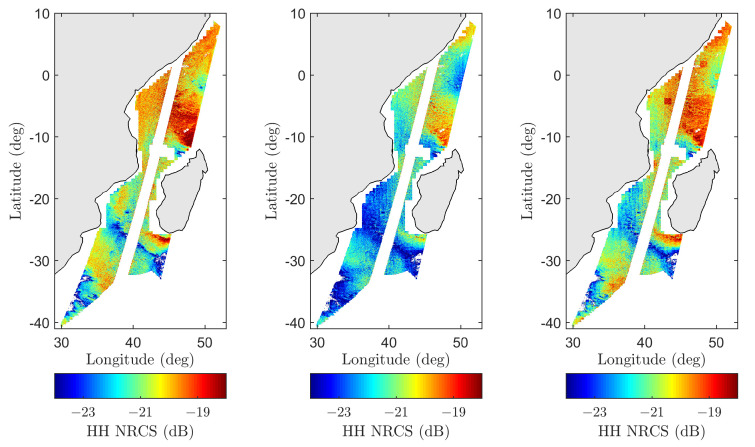
SMAP 1 km high-resolution measured and modeled backscatter NRCS maps for passes 02260_D shown in Figure 16. From (**left**) to (**right**): SMAP, wind-only model, and wind and swell model.

Figure 16 compares SMAP and model predictions for pass 02260_D on 5 July 2015, at 02:32:47 UTC near coastal Madagascar at the glo_30m spatial resolution. Measured and modeled backscatter NRCS maps highlight significant spatial modulations of radar returns, while the ancillary data maps (wind speed, swell-only SWH, and average wave period) highlight the underlying sources of these variations. While the wind-only predictions capture many large-scale features, they largely underestimate the SMAP measurements as expected. The inclusion of swell features, which predominantly occupy the lower half of the scene, significantly improves the overall model and measurement agreement. The prediction improvement is more clearly shown in the scatter plot, where the percentage of predictions within ±1 dB of SMAP measurements improves from 25% to 85%. Figure 17 further shows the corresponding backscatter NRCS measurements and model predictions at the original 1 km SMAP resolution. The high-resolution maps are also consistent with the results shown in Figure 16.

**Figure 18:**
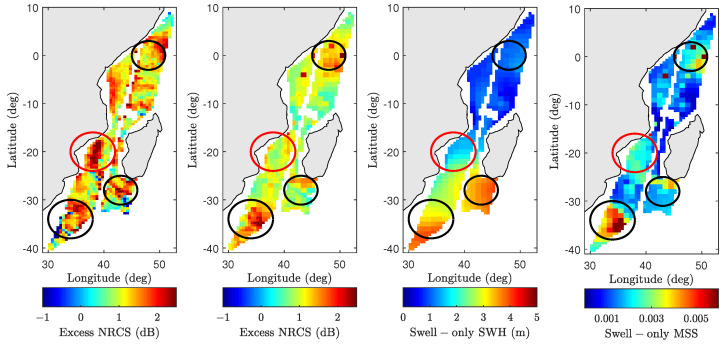
Comparison of swell features in HH-polarized SMAP data for pass 02260_D 5 July 2015, at 02:32:47 UTC. Plots from (**left**) to (**right**) show excess SMAP NRCS (SMAP-TSM (W)), excess model NRCS (TSM (W+S)-TSM (W)), WW3 swell-only SWH, and swell-only MSS derived from approximated 2D spectra based on WW3 partitioned data, respectively.

The improved backscatter NRCS prediction capability under all wind speeds can next be used to identify possible swell features present in the SMAP data. As this study characterizes swell effects as an excess slope contribution, excess NRCS obtained by subtracting wind-only model results from SMAP measurements and model predictions can be compared as shown in Figure 18. The WW3-derived swell-only SWH and numerically computed swell-only MSS are also shown. For ease of comparison, excess NRCS features of particular interest are highlighted using circles along with corresponding locations on swell-only SWH and excess swell-only MSS maps. Features that are captured by the wave model are highlighted in black circles (for example, the swell feature on the bottom left corner of the map). In contrast, the red circle indicates a significant apparent wave effect in the SMAP image that is not well predicted by the model, potentially due to an inaccuracy in the predicted swell properties at this time and location.

The swell only MSS map shown in Figure 18 also indicates the magnitude of swell-only MSS relative to wind-only MSS computed using Equation (3). As expected, low swell-only SWH regions (<1 m) produce negligible swell-only MSS contributions (≈0.001). However, the strong swell feature in the bottom-left corner of the figure (swell-only SWH ≈4 m) results in swell-only MSS values up to 0.018–0.02, which, based on Equation (3), corresponds to a wind speed $u_{19.5}\approx 4\;{\mathrm{ms}}^{-1}$. As seen in the figure, the impact of this is marginal over high-wind regions, but can be significant when the wind speed is very low.

As mentioned in the introduction, the impact of swell on C- and Ku-band instruments has been investigated in the literature. An analysis of ERS-1/2 SAR data in [12] concludes that swell has some impact on C-band backscatter NRCS measurements. While the overall impact appears to minimal over moderate to high wind speeds, Ref. [62] finds wave effects to have an outsize impact under certain localized low-wind/high SWH conditions. This study also finds evidence of greater swell wave impact on L-band backscatter NRCS under similar localized conditions as shown in Figure 16 and Figure 18. Similar conclusions are drawn in [13] where C-band sensitivity to wave effects is somewhat higher compared Ku-band. The 2–2.5 dB mean improvement shown in Figure 15 (though small in absolute terms) indicates a relatively larger impact on L-band backscatter NRCS compared to C- or Ku-band frequencies. This may be due to the larger marginal long-wave MSS contribution at L-band. As MSS is primarily supported by the short waves, the larger cutoff wavenumbers for C- and Ku-bands result in more MSS due to wind-driven waves and lessens the marginal impact of swell waves.

**Figure 19:**
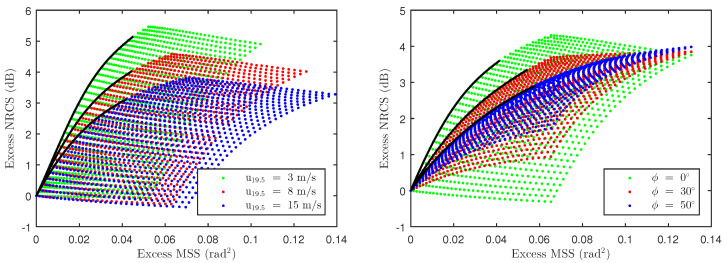
The distribution of excess MSS and NRCS versus wind speed (at ϕ=0°) and relative azimuth (at $u_{10}=10$ m/s) are shown in (**left**) and (**right**) plots, respectively. The leading edge of each surface, approximated using 4th-degree polynomial, is indicated using black curves.

**Figure 20:**
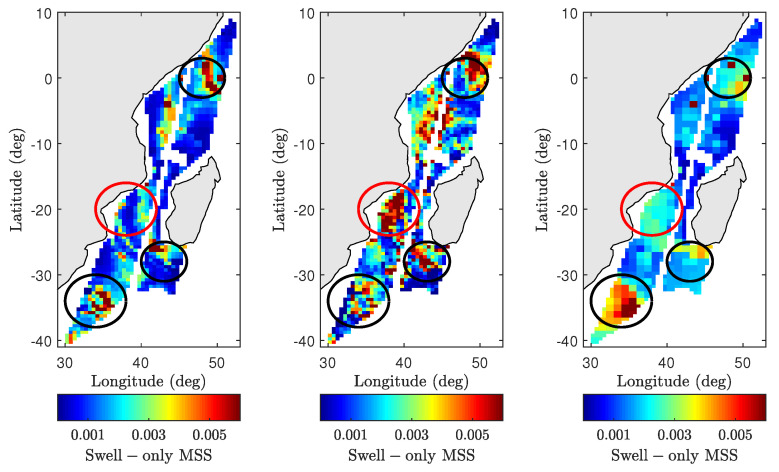
Swell retrieval comparisons for SMAP pass 02260_D. Plots from (**left**) to (**right**) show retrieved excess MSS based on the wind + swell model, retrieved excess MSS based on SMAP data, and excess MSS computed using 2D approximated spectra, respectively. Excess MSS retrievals are based on excess NRCS results shown in Figure 16. See text for more details.

The model developed can be used to examine the inverse problem of retrieving swell properties in the form of an excess MSS map, provided that knowledge of wind vector is available. This is a challenging one-to-many problem where a single excess NRCS value can be mapped to multiple excess MSS values as shown in Figure 19. In order to simplify the estimation process, excess MSS is assumed to be a scalar multiple of the leading-edges of the 2D surfaces in Figure 19. The leading edges are estimated using a 4th-degree polynomial of the form

(40) $$y=a_{0}x^{4}+a_{1}x^{3}+a_{2}x^{2}+a_{3}x$$

where *x* and *y* represent the excess MSS and excess NRCS, respectively. Note that all polynomials traverse through (x,y) = (0,0) indicating no excess NRCS predictions in the absence of excess MSS. The leading-edge definition used above predicts the smallest possible excess MSS for a given excess NRCS observation. As shown in Figure 18, the excess NRCS is first computed by subtracting wind-only TSM predictions from wind + swell model predictions and SMAP observations. Equation (40) can then be inverted iteratively to find an excess MSS such that the resulting excess NRCS is within ±0.01 dB of the computed value above. Figure 20 shows the resulting excess MSS retrievals for wind + swell model (left figure) and SMAP observations (middle figure), while the right figure shows excess MSS computed using 2D swell-only approximated spectra discussed in Section 5.3. Note that features highlighted using circles match those described in Figure 18. The initial results are consistent with excess NRCS maps in Figure 18 as well as the excess swell MSS computed using the 2D approximated spectra.

While these initial results suggest the potential of swell MSS retrieval, the inversion process is challenged by several potential error sources. We have discussed the impact of wind spectrum choice and wave age in preceding sections, but there may also be potential errors in ancillary wind vector information. Furthermore, the near-coastal coverage of the SMAP L1-C dataset makes near-coastal bathymetry an important factor. For example, the spatially varying water depths may introduce additional sources of surface roughness (changing currents, tidal flow, etc.) and may also increase the impact of effects such as breaking waves that generally has a low impact on L-band observations. Therefore, additional work and examples are clearly required for continued progress, and will be considered in future work.

## 7. Conclusions

This paper presented an investigation of the impact of swell waves on NASA’s SMAP L1-C high-resolution SAR backscatter NRCS measurements. The radar data was pre-processed and averaged to GFS/WW3 30 arc-minute spatial resolution to generate a wind-wave-NRCS match-up dataset. Two physical models, namely TSM and SSA2-HF, were used to model the backscatter NRCS in co- and cross-polarizations, respectively. Scatter density plots shown in Figure 9 confirmed the backscatter NRCS dependence on wind speed, but also indicated the non-negligible impact of swell waves on those observations.

The impact of swell waves on backscatter NRCS was modeled as an excess slope contribution that leads to additional tilting of Bragg waves under the TSM. Although various wind-driven spectra are available in the literature (the Durden–Vesecky spectrum was chosen), the study was challenged by the absence of a general swell spectrum model to compute excess swell-only MSS. A detailed analysis of NDBC buoy data was carried out to assess the suitability of the JONSWAP spectrum to be used for swell spectrum modeling. The results successfully extend the JONSWAP parameters γ, $\sigma_{a}$, and $\sigma_{b}$ into the swell regime as shown in Figure 10. The moment parameters associated with WW3 partitioned wave spectra were then used to approximate the 2D swell-only spectra as shown in Figure 11. They were numerically integrated to obtain excess MSS due to swell in up- and cross-wind directions.

These excess MSS values were then incorporated into the TSM to make combined wind and swell backscatter NRCS predictions. The combined model results demonstrated a significant improvement over the conventional wind-only model predictions. Model predictions also identified a disagreement under low wind speeds, which was corrected by modifying the DV spectrum over low wind speeds ($u_{19.5}\leq 10\;{\mathrm{ms}}^{-1}$). The assumed presence of a fully-developed sea surface was also investigated in detail as shown in Figure 13, and the wind + swell model was further modified to accommodate fetch-limited ocean conditions.

The refined results show a significant improvement in backscatter NRCS prediction capability. As hypothesized, a larger improvement in HH-polarized backscatter NRCS predictions was observed compared to VV, while the cross-polarized observations were limited by system noise. In general, the impact of swell waves was more prominent under low wind conditions. Although small in absolute terms, the impact of swell on L-band backscatter NRCS appears to be larger compared higher radar frequencies. The excess NRCS features were then examined carefully for the presence of swell features, and the results demonstrate the modeling capability achieved. The inverse problem of predicting swell (excess MSS) based on excess NRCS was also examined. Even though the initial results appeared promising, the swell prediction process should be further refined to make more broadly applicable conclusions.

## Figures and Tables

**Table 1:** SMAP L1-C high-resolution data summary (year: 2015; revision: R13080_001). Downloaded from Alaska Satellite Facility [49].

Month	Start Orbit	End Orbit	# of Data Files	Size (GB)
04	01056_A	01308_A	504	650.8
05	01308_D	01761_D	837	1251.2
06	01762_A	02200_A	785	1198.6
07	02200_D	02301_D	200	308.8

**Table 2:** An example of partitioned spectrum data. Total wavefield, wind-sea and swell-sea information are provided by partitions 0, 1, and 2–4, respectively. For each partition, $H_{1/3}$: total significant wave height in meters; $T_{p}$: peak period in seconds; $\Lambda_{p}$: wavelength at peak period; $\phi_{m}$: mean wave direction at peak period in degrees; $\sigma_{\phi }$: spectral width in degrees; and $W_{f}$: wind-sea fraction.

Partition # $P_{n}$	$H_{1/3}$ (m)	$T_{p}$ (s)	$\Lambda_{p}$ (m)	$\phi_{m}$ (deg)	$\sigma_{\phi }$ (deg)	$W_{f}$ (-)
0	2.91	11.28	198.59	325.99	33.22	0.13
1	2.80	11.55	208.36	326.48	24.49	0.15
2	0.62	9.21	132.51	1.83	6.94	0
3	0.37	13.72	293.87	191.07	10.12	0
4	0.34	11.03	189.83	193.14	7.73	0

## Data Availability

All datasets used in this study are discussed in Section 3. They are publicly available and cited in the list of references.

## References

[1] Apel J.R. (1999). Principles of Ocean Physics.

[2] Dickey T.D. (1991). The Emergence of Concurrent High-Resolution Physical and Bio-Optical Measurements in the Upper Ocean and their Applications. Rev. Geophys..

[3] Wright J. (1968). A New Model for Sea Clutter. IEEE Trans. Antennas Propag..

[4] Bryan K. (1969). Climate and the Ocean Circulation 3: The Ocean Model. Mon. Weather Rev..

[5] Tolman H.L., Accensi M., Alves J.H., Ardhuin F., Barbariol F., Benetazzo A., Bennis A.C., Bidlot J., Booij N., Boutin G. (2016). User Manual and System Documentation of WAVEWATCH III (R) Version 5.16.

[6] (2018). Part VII: ECMWF Wave Model. IFS Documentation CY45R1.

[7] Zhang J., Wang W., Guan C. (2011). Analysis of the Global Swell Distributions using ECMWF Re-analyses Wind Wave Data. J. Ocean Univ. China.

[8] Zheng K., Sun J., Guan C., Shao W. (2016). Analysis of the Global Swell and Wind Sea Energy Distribution Using WAVEWATCH III. Adv. Meteorol..

[9] Gjevik B., Rygg O., Krogstad H.E., Lygre A. (1988). Long Period Swell Wave Events on the Norwegian Shelf. J. Phys. Oceanogr..

[10] Chen G., Chapron B., Ezraty R., Vandemark D. (2002). A Global View of Swell and Wind Sea Climate in the Ocean by Satellite Altimeter and Scatterometer. J. Atmos. Ocean. Technol..

[11] Collard F., Ardhuin F., Chapron B. (2008). Persistency of Ocean Swell Fields Observed from Space. arXiv.

[12] Guo J., He Y., Perrie W., Shen H., Chu X. (2009). A New Model to Estimate Significant Wave Heights with ERS-1/2 Scatterometer Data. Chin. J. Oceanol. Limnol..

[13] Hwang P.A., Plant W.J. (2010). An Analysis of the Effects of Swell and Surface Roughness Spectra on Microwave Backscatter from the Ocean. J. Geophys. Res. Oceans.

[14] Valenzuela G.R. (1978). Theories for the Interaction of Electromagnetic and Oceanic Waves—A Review. Bound.-Layer Meteorol..

[15] Hasselmann K., Raney R.K., Plant W.J., Alpers W., Shuchman R.A., Lyzenga D.R., Rufenach C.L., Tucker M.J. (1985). Theory of synthetic aperture radar ocean imaging: A MARSEN view. J. Geophys. Res. Oceans.

[16] Swift C., Wilson L. (1979). Synthetic Aperture Radar Imaging of Moving Ocean Waves. IEEE Trans. Antennas Propag..

[17] Alpers W.R., Ross D.B., Rufenach C.L. (1981). On the Detectability of Ocean Surface Waves by Real and Synthetic Aperture Radar. J. Geophys. Res. Oceans.

[18] Hasselmann K., Hasselmann S. (1991). On the Nonlinear Mapping of an Ocean Wave Spectrum into a Synthetic Aperture Radar Image Spectrum and its Inversion. J. Geophys. Res. Oceans.

[19] Evans D.L., Apel J., Arvidson R., Bindschadler R., Carsey F., Dozier J., Jezek K., Kasischke E., Li F., Melack J. (1995). Spacebourne Synthetic Aperture Radar: Current Status and Future Directions.

[20] Entekhabi D., Njoku E.G., O’Neill P.E., Kellogg K.H., Crow W.T., Edelstein W.N., Entin J.K., Goodman S.D., Jackson T.J., Johnson J. (2010). The Soil Moisture Active Passive (SMAP) Mission. Proc. IEEE.

[21] Wijesundara S.N., Johnson J.T. Swell Effects on Near-Coastal Smap L-Band High-Resolution NRCS Data. Proceedings of the 2019 IEEE International Geoscience and Remote Sensing Symposium (IGARSS).

[22] Valenzuela G.R. (1968). Scattering of Electromagnetic Waves From a Tilted Slightly Rough Surface. Radio Sci..

[23] Plant W.J. (1986). A Two-Scale Model of Short Wind-Generated Waves and Scatterometry. J. Geophys. Res. Oceans.

[24] Johnson J.T., Shin R.T., Kong J.A., Tsang L., Pak K. (1998). A Numerical Study of the Composite Surface Model for Ocean Backscattering. IEEE Trans. Geosci. Remote Sens..

[25] Elfouhaily T.M., Guérin C.A. (2004). A Critical Survey of Approximate Scattering Wave Theories from Random Rough Surfaces. Waves Random Media.

[26] Cox C., Munk W. (1954). Measurement of the Roughness of the Sea Surface from Photographs of the Sun’s Glitter. J. Opt. Soc. Am..

[27] Durden S.L., Vesecky J.F. (1990). A Numerical Study of the Separation Wavenumber in the Two-Scale Scattering Approximation. IEEE Trans. Geosci. Remote Sens..

[28] Brown G. (1978). Backscattering From a Gaussian-Distributed Perfectly Conducting Rough Surface. IEEE Trans. Antennas Propag..

[29] Lyzenga D. Effects of wave breaking on SAR signatures observed near the edge of the Gulf Stream. Proceedings of the 1996 International Geoscience and Remote Sensing Symposium (IGARSS’96).

[30] Voronovich A.G., Zavorotny V.U. (2001). Theoretical model for scattering of radar signals in Ku- and C-bands from a rough sea surface with breaking waves. Waves Random Media.

[31] Hwang P.A., Fois F. (2015). Surface roughness and breaking wave properties retrieved from polarimetric microwave radar backscattering. J. Geophys. Res. Oceans.

[32] Voronovich A.G. (1994). Wave Scattering from Rough Surfaces.

[33] Guérin C., Johnson J.T. (2015). A Simplified Formulation for Rough Surface Cross-Polarized Backscattering Under the Second-Order Small-Slope Approximation. IEEE Trans. Geosci. Remote Sens..

[34] Pierson W.J., Moskowitz L. (1964). A Proposed Spectral Form for Fully Developed Wind Seas Based on the Similarity Theory of S. A. Kitaigorodskii. J. Geophys. Res..

[35] Hasselmann K., Barnett T.P., Bouws E., Carlson H., Cartwright D.E., Enke K., Ewing J.A., Gienapp H., Hasselmann D.E., Kruseman P. (1973). Measurements of Wind-Wave Growth and Swell Decay during the Joint North Sea Wave Project (JONSWAP).

[36] Durden S., Vesecky J. (1985). A Physical Radar Cross-Section Model for a Wind-Driven Sea with Swell. IEEE J. Ocean. Eng..

[37] Elfouhaily T., Chapron B., Katsaros K., Vandemark D. (1997). A Unified Directional Spectrum for Long and Short Wind-driven Waves. J. Geophys. Res. Oceans.

[38] Yurovskaya M.V., Dulov V.A., Chapron B., Kudryavtsev V.N. (2013). Directional Short Wind Wave Spectra Derived from the Sea Surface Photography. J. Geophys. Res. Oceans.

[39] Ryabkova M., Karaev V., Guo J., Titchenko Y. (2019). A Review of Wave Spectrum Models as Applied to the Problem of Radar Probing of the Sea Surface. J. Geophys. Res. Oceans.

[40] Hwang P.A., Ainsworth T.L. (2020). L-Band Ocean Surface Roughness. IEEE Trans. Geosci. Remote Sens..

[41] Zhou X., Chong J., Yang X., Li W., Guo X. (2017). Ocean Surface Wind Retrieval using SMAP L-Band SAR. IEEE J. Sel. Top. Appl. Earth Obs. Remote Sens..

[42] Yueh S.H., Tang W., Fore A.G., Neumann G., Hayashi A., Freedman A., Chaubell J., Lagerloef G.S.E. (2013). L-Band Passive and Active Microwave Geophysical Model Functions of Ocean Surface Winds and Applications to Aquarius Retrieval. IEEE Trans. Geosci. Remote Sens..

[43] Yueh S.H., Chaubell J. (2012). Sea Surface Salinity and Wind Retrieval Using Combined Passive and Active L-Band Microwave Observations. IEEE Trans. Geosci. Remote Sens..

[44] Isoguchi O., Shimada M. (2009). An L-Band Ocean Geophysical Model Function Derived From PALSAR. IEEE Trans. Geosci. Remote Sens..

[45] Yueh S.H. (1997). Modeling of Wind Direction Signals in Polarimetric Sea Surface Brightness Temperatures. IEEE Trans. Geosci. Remote Sens..

[46] Phillips O.M. (1957). On the Generation of Waves by Turbulent Wind. J. Fluid Mech..

[47] West R. (2012). Soil Moisture Active Passive (SMAP), Algorithm Theoretical Basis Document (ATBD), SMAP Level 1 Radar Data Products.

[48] Weiss B., Madatyan M. (2016). Soil Moisture Active Passive (SMAP) Project Level 1C_S0_HiRes Product Specification Document Revision C.

[49] SMAP Data 2015 (NASA) Dataset: SMAP_L1C_S0_HiRes_V3. https://asf.alaska.edu/.

[50] Tracy B., Devaliere E., Hanson J., Nicolini T., Tolman H. Wind Sea and Swell Delineation for Numerical Wave Modeling. Proceedings of the 10th International Workshop on Wave Hindcasting and Forecasting & Coastal Hazard Symposium.

[51] Chawla A., Cao D., Gerald V.M., Spindler T., Tolman H.L. Operational Implementation of a Multi-grid Wave Forecast System. Proceedings of the 10th International Workshop on Wave Hindcasting and Forecasting & Coastal Hazerd Symposium.

[52] US DOC/NOAA/NWS/NDBC > National Data Buoy Center (1971) Meteorological and Oceanographic Data Collected from the National Data Buoy Center Coastal-Marine Automated Network (C-MAN) and Moored (Weather) Buoys. Subset 2014/2015 05-07. NOAA National Centers for Environmental Information. Dataset. https://www.ncei.noaa.gov/archive/accession/NDBC-CMANWx.

[53] Montoya R., Osorio Arias A., Ortiz Royero J., Ocampo-Torres F. (2013). A wave parameters and directional spectrum analysis for extreme winds. Ocean Eng..

[54] Lucas C., Soares C.G. (2015). On the Modelling of Swell Spectra. Ocean Eng..

[55] Hanson J.L., Phillips O.M. (2001). Automated Analysis of Ocean Surface Directional Wave Spectra. J. Atmos. Ocean. Technol..

[56] Wang D.W., Hwang P.A. (2001). An Operational Method for Separating Wind Sea and Swell from Ocean Wave Spectra. J. Atmos. Ocean. Technol..

[57] Portilla J., Ocampo-Torres F.J., Monbaliu J. (2009). Spectral Partitioning and Identification of Wind Sea and Swell. J. Atmos. Ocean. Technol..

[58] Hwang P.A., Ocampo-Torres F.J., García-Nava H. (2012). Wind Sea and Swell Separation of 1D Wave Spectrum by a Spectrum Integration Method. J. Atmos. Ocean. Technol..

[59] Kuik A.J., van Vledder G.P., Holthuijsen L.H. (1988). A Method for the Routine Analysis of Pitch-and-Roll Buoy Wave Data. J. Phys. Oceanogr..

[60] Zavorotny V.U. (2019). WW3 MSS Extension up to the L-Band Cutoff.

[61] Wang T., Zavorotny V.U., Johnson J., Ruf C., Yi Y. Modeling of Sea State Conditions for Improvement of Cygnss L2 Wind Speed Retrievals. Proceedings of the 2018 IEEE International Geoscience and Remote Sensing Symposium (IGARSS 2018).

[62] Quilfen Y., Chapron B., Collard F., Vandemark D. (2004). Relationship between ERS Scatterometer Measurement and Integrated Wind and Wave Parameters. J. Atmos. Ocean. Technol..

